# Novel Fast Chromatography-Tandem Mass Spectrometric Quantitative Approach for the Determination of Plant-Extracted Phytosterols and Tocopherols

**DOI:** 10.3390/molecules26051402

**Published:** 2021-03-05

**Authors:** George Gachumi, Alice Demelenne, Asmita Poudel, Zafer Dallal Bashi, Anas El-Aneed

**Affiliations:** 1 Drug Design and Discovery Group, College of Pharmacy and Nutrition, University of Saskatchewan, Saskatoon, SK S7N 5E5, Canada; george.gachumi@usask.ca (G.G.); asp170@mail.usask.ca (A.P.); zafer.bashi@usask.ca (Z.D.B.); 2 Laboratory for the Analysis of Medicines, Department of Pharmacy, CIRM, University of Liège, 4000 Liege, Belgium; alice.demelenne@uliege.be

**Keywords:** phytosterols, tocopherols, fast chromatography-mass spectrometry, canola deodorizer distillate

## Abstract

Phytosterols and tocopherols are commonly used in food and pharmaceutical industries for their health benefits. Current analysis methods rely on conventional liquid chromatography, using an analytical column, which can be tedious and time consuming. However, simple, and fast analytical methods can facilitate their qualitative and quantitative analysis. In this study, a fast chromatography-tandem mass spectrometric (FC-MS/MS) method was developed and validated for the quantitative analysis of phytosterols and tocopherols. Omitting chromatography by employing flow injection analysis—mass spectrometry (FIA-MS) failed in the quantification of target analytes due to analyte-to-analyte interferences from phytosterols. These interferences arise from their ambiguous MS fingerprints that would lead to false identification and inaccurate quantification. Therefore, a C18 guard column with a 1.9 µm particle size was employed for FC-MS/MS under isocratic elution using acetonitrile/methanol (99:1 *v*/*v*) at a flow rate of 600 µL/min. Analyte-to-analyte interferences were identified and eliminated. The false peaks could then be easily identified due to chromatographic separation. In addition, two internal standards were evaluated, namely cholestanol and deuterated cholesterol. Both internal standards contributed to the observed analyte-to-analyte interferences; however, adequate shift in the retention time for deuterated cholesterol eliminated its interferences and allowed for an accurate quantification. The method is fast (1.3 min) compared to published methods and can distinguish false peaks observed in FIA-MS. Seven analytes were quantified simultaneously, namely brassicasterol, campesterol, stigmasterol, β-sitosterol, α-tocopherol, δ-tocopherol, and γ-tocopherol. The method was successfully applied in the quantitative analysis of phytosterols and tocopherols present in the unsaponifiable matter of canola oil deodorizer distillate (CODD). β-sitosterol and γ-tocopherol were the most abundant phytosterols and tocopherols, respectively.

## 1. Introduction

**Figure 1:**
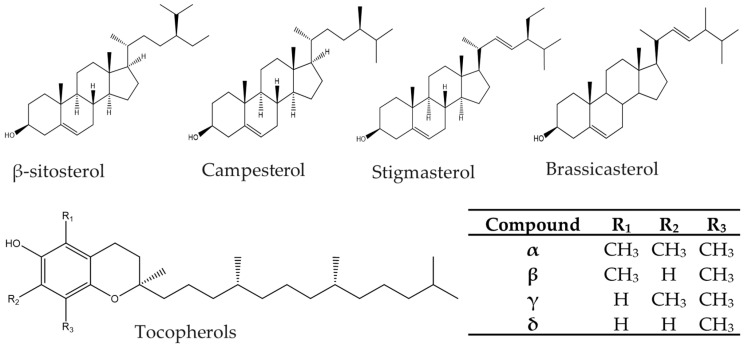
Structures of abundant phytosterols and tocopherols (α-alpha, β-beta, γ-gamma, and δ-delta).

Phytosterols are plant derived sterols with established cholesterol lowering properties, while tocopherols possess vitamin E activity and act as natural antioxidants [1,2,3]. Due to these health benefits, their incorporation in functional foods is approved by the U.S Food and Drug Administration (FDA) and Commission Regulation (EU) [4,5], and are commonly used as dietary supplements and food additives. The composition and abundance of phytosterols and tocopherols vary among various plant sources but are widely obtained from vegetable oils, such as soybean, canola, palm, sunflower, corn, and olive oils [6,7]. Phytosterols and tocopherols are part of the unsaponifiable matter. They are isolated in pure form via the application of various purification techniques such as liquid-liquid extraction, solid phase extraction or crystallization [8,9,10]. Abundant phytosterols in vegetable oils are β-sitosterol, campesterol, stigmasterol, and brassicasterol (unique in brassica oils, such as rapeseed oil), and four isoforms of tocopherols, namely alpha (α), beta (β), gamma (γ), and delta (δ) (Figure 1). Consumers have become health-conscious, driving the demand for natural bioactives relative to their synthetic counterparts. Consequently, the use of natural products as food additives is favored. Regardless of the source for the bioactives, a fast, simple, and sensitive analytical method is required for their identification and quantification [11,12]. Traditionally, gas chromatography (GC) with flame ionization detector (FID) [13,14,15,16,17] or mass spectrometry (MS) [18,19,20,21] has been utilized for the determination phytosterols and tocopherols. However, derivatization of analytes is usually required in GC to improve analytes’ volatility, thermal stability, and sensitivity [22]. Despite its separation capability including the ability to resolve structural isomers [22,23], GC is laborious, requiring long run time and is not ideal for routine analysis.

To avoid derivatization, liquid chromatography-mass spectrometry (LC-MS) has been adapted. Ionization at atmospheric pressure is employed and both electrospray ionization (ESI) and atmospheric pressure chemical ionization (APCI) have been used for the analysis of phytosterols and tocopherols [24,25,26]. Although tocopherols ionize efficiently in both ESI and APCI, phytosterols ionize better using APCI [27,28,29]. Phytosterols primarily ionize as [M + H − H_2_O]^+^ ion while [M + H]^+^ and [M + H − 4H]^+^ appear as minor ions in both APCI and ESI [30,31]. Tocopherols, on the other hand, ionize in APCI as the protonated species [M + H]^+^ as well as the molecular ion [M]^+^ [32,33,34]. Both normal phase (NP) and reversed phase (RP) chromatography have been used for the analysis of phytosterols and tocopherols. The former showed better separation, particularly the ability to resolve the structural isomers, β and γ-tocopherols [15]. However, the introduction of columns that combine hydrophobic, charge transfer, and π–π interactions such as pentafluorophenyl (PFP) have enabled the application of RP-LC that shows baseline separation of the four tocopherol isoforms [35,36].

Numerous LC-MS methods have been developed for the analysis of phytosterols [37,38], tocopherols [35,36], or their combined mixture [24,34]. Simultaneous determination of phytosterols and tocopherols is ideal when the two group of analytes should be quantified as it simplifies the analytical procedure. Recently, we developed LC-MS quantification and screening methods for the simultaneous analysis of phytosterols and tocopherols with an analysis run time of 6.5 min [39,40]. An alternative strategy for fast quantitative analysis is flow injection analysis (FIA)-MS, that involves the omission of the analytical column, provided that the robustness of quantification is not compromised [41,42,43]. Although “separation” in case of FIA-MS relies on the high selectivity of MS, such ability is lost when more than one analyte shares the exact precursor ion (e.g., isomers, isobars) in the single ion monitoring (SIM) mode or have similar transitions when employing multiple reaction monitoring (MRM). In fact, quantitative FIA-MS is commonly reported for one or two analytes and is challenging when analyzing multiple targets [44]. FIA-MS is, therefore, not generally suitable for the analysis of isomers or structurally similar compounds as selectivity is compromised. MS suffers from analyte-to-analyte interferences that result in ions with similar *m*/*z* value or mass transitions with identical product ions. These interferences in FIA-MS originate from in-source fragmentations and they can undermine the analytical results [45,46]. However, they can be easily identified by analyzing pure individual reference standards [47].

Analyte-to-analyte interferences for phytosterols have already been reported (supplementary materials Table S1). For example, during MS analysis, campesterol, which was monitored at *m*/*z* 383 as [M + H − H_2_O]^+^ ion, also produced during ionization two ions at *m*/*z* 395 and 397 that matches the [M + H − H_2_O]^+^ of stigmasterol and sitosterol, respectively. Similarly, sitosterol, which was monitored at *m*/*z* 397 as [M + H − H_2_O]^+^ ion, produced an ion at *m*/*z* 409 that have the same m/z value of [M + H − H_2_O]^+^ of cycloartenol [48]. Naoyuki Ishida reported the monitoring of stigmasterol [M + H − 4H]^+^ at *m*/*z* 409.4 instead of [M + H − H_2_O]^+^ at *m*/*z* 395.4 due to peak overlap (interference) with campesterol [49]. Despite reporting these interferences, the exact structures for the various interfering ions was never fully elucidated with few studies hypothesizing that they arise from either dehydrogenation or cyclization of the parent compound [50,51]. Unlike phytosterols, no interferences have been reported during the analysis of tocopherols.

To achieve fast analysis without compromising selectivity, fast chromatography (FC), using a guard column only, can allow for sufficient separation in a short time in comparison to conventional chromatography (using an analytical column) [44,52]. In this study, we report for the first time, the development of a FC-MS/MS method for the simultaneous analysis of phytosterols and tocopherols with a total run time of 1.3 min. In addition, the ambiguous MS fingerprints of phytosterols and how they contribute to analyte-to-analyte interferences were identified, discussed and eliminated. Although the intrinsic capabilities of mass spectrometer can be employed in analysis, understanding the MS fingerprints of analytes is crucial. The reported work provides novel strategies for measuring target analytes and expands the knowledge base regarding the reliance on the intrinsic capabilities of mass spectrometer for validated quantitative analysis.

## 2. Results and Discussion

### 2.1. Method Development

We previously developed and reported LC-APCI-MS/MS method for the simultaneous analysis of phytosterols and tocopherols, including their ionization and fragmentation behavior [39,40]. Analyte-to-analyte interferences amongst phytosterols were identified; however, due to the use of conventional chromatography with an analytical column, they did not interfere with identification and quantification. Chromatographic separation of the analytes, allowed for identification and quantification, despite the presence of interfering peaks at different retention times. To the best of our knowledge, no current study has succinctly explained the analyte-to-analyte interferences and their effects during the analysis of phytosterols. Understanding these interferences will allow for the development of rapid analytical methods, such as FIA- or FC-MS that can substantially reduce the analysis time. In this work, the sources of interferences were identified as the minor ions [M + H − 2H]^+^ and [M + H − 4H]^+^ (Table 1). These interfering ions matches the *m*/*z* value of another analyte monitored as [M + H − H_2_O]^+^. For example, brassicasterol which is monitored at *m*/*z* 381 as [M + H − H_2_O]^+^ will also ionize as [M + H − 4H]^+^ at *m*/*z* 395 which is the same *m*/*z* value for the [M + H − H_2_O]^+^ ion for stigmasterol, while campesterol which is monitored at *m*/*z* 383 as [M + H − H_2_O]^+^ will also ionize as [M + H − 4H]^+^ at *m*/*z* 397 which is the same *m*/*z* value for the [M + H − H_2_O]^+^ ion for β-sitosterol (Table 1). Thus, brassicasterol ionization produces a “false” stigmasterol peak while campesterol ionization yields a “false” β-sitosterol peak.

Although the ion designated [M + H − 2H]^+^ was also observed for all the reference standards, only the ion [M + H − 4H]^+^ contributed to the observed interferences when [M + H − H2O]^+^ is selected as the precursor ion in the MRM mode. In addition, cholestanol, phytosterols’ internal standard (IS) also contributed interfering ions for brassicasterol at *m*/*z* 381→147 and campesterol at *m*/*z* 383→161 and 383→147 (Supplementary materials Figure S1). The formation of such ions from the IS is unclear since they did not arise from the corresponding minor ions [M + H − 2H]^+^ and [M + H − 4H]^+^ (Table 1). All the identified minor ions are structurally distinct from that of the actual compound; however, the MS/MS fragmentation patterns are similar. The monitored ion [M + H − H_2_O]^+^ and the corresponding interfering minor ions with the same *m*/*z* value show similar product ions and cannot be differentiated (Supplementary Materials Figures S2–S4). Thus, even when a different product ion is selected, the interferences are not eliminated due to the similarities in product ions of the actual compound and those from minor ions. It should be noted that the interfering ion in case of the IS is not seen at the retention time of the campesterol in case of LC-MS [39] indicating that it is not a contamination within the IS itself.

When FIA-MS/MS is employed for the simultaneous analysis of phytosterols and tocopherols, erroneous identification and quantification of phytosterols are observed due to the interferences described above. The MRM transitions of the monitored target compounds and those from interferences are identical and this issue can only be resolved by utilizing some level of chromatographic separation; hence, we adopted the fast chromatography approach as discussed below.

#### 2.1.1. Fast Chromatography (FC)

To address the issue of analyte-to-analyte interferences for phytosterols and maintain a fast analysis time, the use of a guard column is evaluated. The goal is to achieve sufficient chromatographic separation that can differentiate the signal for the analyte of interest from the interfering signal.

**Figure 2:**
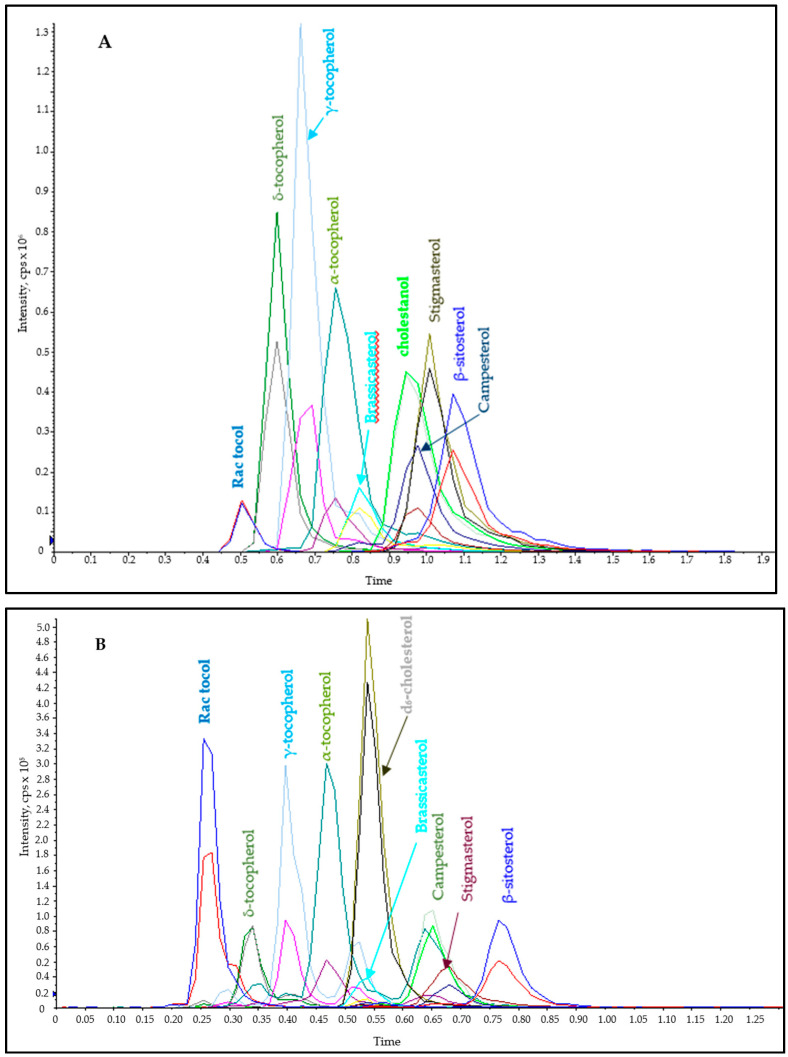
FC-MS/MS chromatogram of phytosterols and tocopherols on (**A**) 2.7 µm guard column at 200 µL/min and (**B**) 1.9 µm guard column at 600 µL/min. Better separation of analytes including the interfering ions was achieved by using1.9 µm guard column.

A C18 poroshell guard column (2.1 mm × 5 mm, 2.7 µm) was used and its performance was evaluated based on its ability to distinctly resolve the peaks of interest from interfering peaks. An isocratic elution with acetonitrile/methanol 99:1 (*v*/*v*) was employed at a flow rate of 200 µL/min. In addition, column temperature was varied between 10–20 °C with better separation achieved at 10 °C, and this was applied throughout the analysis. The total run time at the optimized condition was 2 min. Although enough separation was achieved to differentiate the interfering peaks, brassicasterol and campesterol coeluted closely with cholestanol (IS) (Figure 2A). As already mentioned, this IS contributed to interferences for both campesterol and brassicasterol. At low concentrations, these interfering peaks were apparent as a shoulder peak; however, at higher concentrations they were completely merged with the analyte of interest thereby compromising the analytical results (Supplementary materials Figure S5). In fact, in the presence of internal standard cholestanol, campesterol cannot be resolved.

To address the interference from cholestanol, the IS was substituted with another compound, namely deuterated cholesterol (d_6_) (Table 1). However, d_6_-cholesterol contributed to the formation of an interfering peak that matches campesterol (Supplementary materials Figure S6) and since they closely coeluted, the interfering peak could not be differentiated as it merged with the actual peak for campesterol. As explained earlier about cholestanol interfering peaks, d_6_-cholesterol interfering ion does not match [M + H − 2H]^+^ or [M + H − 4H]^+^ and it possibly arise via a different ionization mechanism.

Further improvements in chromatographic separation are, therefore, needed to address the interference observed from the IS. Chromatographic separation relies on column efficiency, a factor expressed as theoretical plates or height equivalent theoretical plates (H) when normalized with column length [53]. Thus, the more efficient a column is, the smaller is the H term. Stationary phase particle size and morphology are known to play a key role in increasing theoretical plates (equivalent to low H) [54]. To this end, the guard column length had to be maintained for short analysis time and seek a stationary phase of smaller particle size for improved column efficiency. A poroshell C18 guard column with smaller particle size of 1.9 µm was, therefore, tested. The chromatographic conditions were the same as optimized for the 2.7 µm guard column, except for the flow rate which was 600 µL/min and the total run time was 1.3 min.

The results showed a significant improvement when compared to the guard column with a 2.7 µm particle size (Figure 2B). Efficient separation was achieved for all compounds except two pairs that coeluted, namely d_6_-cholesterol/brassicasterol, and campesterol/stigmasterol. However, since the monitored MRMs are different, method selectivity and sensitivity were attained. In fact, d_6_-cholesterol eluted closely with campesterol and the interfering peak was resolved even at high concentrations (Supplementary materials Figure S7). The use of a guard column with 1.9 µm particle size was, therefore, ideal for the simultaneous analysis of phytosterols and tocopherols.

#### 2.1.2. MS Conditions

The ion source and compound dependent parameters were optimized through direct infusion as already reported [39]. Phytosterols ionized as [M + H − H_2_O]^+^, whereas tocopherols showed abundant molecular ion M**^.+^**. For quantification, the MRM mode was selected, and two transitions were monitored for each analyte, quantifier and a qualifier ion (Table 2).

Dwell time, the time needed for acquiring a specific MRM during each cycle can affect the quantitative data. High dwell time is normally desirable for better signal-to-noise ratio; however, it becomes impractical to assign high dwell times for multi analytes coeluting when a short analysis time is desired. A scheduled MRM (sMRM) approach was, therefore, adapted. sMRM algorithm automatically adjusts the dwell time of each compound depending on user defined target scan time (t_Target_). Optimization of t_Target_ was conducted with an aim of achieving at least 10 data points and t_Target_ values of 0.3–0.6 s were tested. A t_Target_ of 0.3–0.4 s allowed for acquiring more data points but it led to distorted chromatographic peak shape, while values above 0.6 s did not provide the minimum number of 8 data points across the chromatographic peak. Therefore, a value of 0.55 s was optimal since it allowed for the acquisition of the minimum data points with good peak shapes (Figure 2B). To further enhance the reproducibility of the method, MRM detection window was also optimized. This window is an estimate of the chromatographic peak width and its value should also accommodate any chromatographic shifts. An MRM detection window of 35 s was optimal, and it was based on the chromatographic peak with largest peak width.

### 2.2. Method Validation

The method validation was conducted following the International Council for Harmonization of Technical Requirements for Pharmaceuticals for Human Use (ICH) guidelines [55], and included linearity, sensitivity, accuracy, precision, repeatability, matrix effects, and recovery.

#### 2.2.1. Calibration Range and Sensitivity

Phytosterols and tocopherols calibration curves were generated by applying a 1/x weighted linear regression analysis in the concentration range 0.05–10 µg/mL. Phytosterols showed a linear regression in the range 0.05–10 µg/mL while tocopherols showed linearity in the range of 0.25–10 µg/mL with a correlation coefficients (R^2^) of at least 0.99 for all analytes. The exception, however, were gamma and delta tocopherols that were fitted to a quadratic calibration curve with a R^2^ of at least 0.99. A quadratic calibration curve for tocopherols was reported previously [39]. Supplementary Materials Figure S8 shows the calibration curves for phytosterols and tocopherols.

The LLOQ value was chosen as the lowest concentration on the calibration curve with an accuracy and precision value within ±20% and were, 0.05 µg/mL and 0.25 µg/mL for phytosterols and tocopherols, respectively. Limit of detection (LOD) for all analytes was determined at the lowest detectable concentrations at S/N ≥ 3. The determined LOD values were 0.005 µg/mL for campesterol and β-sitosterol, 0.01 µg/mL for δ-tocopherol, brassicasterol, and stigmasterol, and 0.05 µg/mL for α- and γ-tocopherol. The reported LLOQ values were similar to previously developed LC-MS/MS method [39]. On the other hand, a two-fold decrease in brassicasterol and δ-tocopherol and five-fold decrease in α- and γ-tocopherol were observed while the LOD values remained the same for campesterol, stigmasterol, and sitosterol.

#### 2.2.2. Intra and Interday Accuracy and Precision

To establish the repeatability of the developed method, accuracy and precision were evaluated on a single day (intraday) and on three different days (interday) at four QC levels (Table 3). QC samples spiked in the matrix were only monitored for δ-tocopherol (LLOQ, LQC, MQC, and HQC) and stigmasterol (MQC, and HQC) as highlighted in Section 3.5 and Section 3.5.2. Four replicates were analyzed at each QC level and all the values were found to be within the acceptable values of accuracy and precision within ±15% CV except at LLOQ which can be ±20% CV for QCs made in neat and those spiked in matrix (Table 3). In fact, over 90% of the QC samples had precision values within ±5% CV from the nominal concentration while the rest were within ±10%. The reported method is developed to measure analytes within vegetable oil samples and the QC samples prepared in the matrix in the case of delta tocopherol and stigmasterol are representatives for tocopherols and phytosterols, respectively. We adopted established methodologies for method validation in the case where analyte-free matrix is not available [56,57].

#### 2.2.3. Recovery and Matrix Effects

Matrix effects were evaluated at both LQC and HQC using δ-tocopherol and stigmasterol. Matrix effects for stigmasterol was only conducted at HQC since its endogenous amount is above the LQC value. Matrix effect for δ-tocopherol at LQC and HQC and stigmasterol at HQC are shown in Table 4. It can be observed that, there is slight ion suppression for δ-tocopherol at HQC and ion enhancement at LQC, and HQC for δ-tocopherol and stigmasterol, respectively. Recovery experiments showed practically no sample loss for stigmasterol while very minor loss (below 5%) was observed for δ-tocopherol.

#### 2.2.4. Stability

Both the benchtop and autosampler QCs used for evaluating stability met the acceptable limits for both accuracy and precision. Thus, the analytes are stable during sample preparation and analysis (Supplementary materials Table S2).

### 2.3. Application of the Method for the Quantification of Phytosterols and Tocopherols

The validated FC-MS method was applied in the quantitative analysis of phytosterols and tocopherols present in the unsaponifiable matter of CODD. For the analyzed samples, the unsaponifiable content in CODD was in the range 26–29%. Two CODD sources were utilized and they differed in the approach employed during the canola oil pressing process, that is, hot pressed or cold pressed. Thus, a total of three samples were analyzed, namely cold pressed CODD and two different batches from hot pressed CODD. As shown in Table 5, six bioactives were identified and quantified in all CODD samples. The composition of these bioactives were α-, and γ-tocopherols, brassicasterol, campesterol, stigmasterol, and β-sitosterol. γ-tocopherol and β-sitosterol were the abundant tocopherol and phytosterol, respectively, while δ-tocopherol was not detected. In addition, stigmasterol was only present in very low concentration and phytosterols abundance were as follows; β-sitosterol > campesterol > brassicasterol > stigmasterol. Phytosterols and tocopherols content in CODD unsaponifiable matter were 30–35% and 2–4%, respectively. All the samples have similar composition and the differences in quantity can be attributed to variations in cultivar, extraction, and refining procedures.

The quantification of phytosterols and tocopherols from vegetable oil samples will provide crucial information in determining the ideal sample to use for extracting these bioactives for commercial use. The final utilization of phytosterols and tocopherols requires their purification from the unsaponifiable matter and therefore, information on the composition and their quantity will be informative in designing efficient extraction procedures.

## 3. Materials and Methods

### 3.1. Reagents and Chemicals

β-sitosterol (Cat. no. S497050), campesterol (Cat. no. C155360), stigmasterol (Cat. no. S686750), brassicasterol (Cat. no. B686750), and d_6_-cholesterol (Cat. no. C432502) at 98% purity were purchased from Toronto Research Chemicals (North York, ON, Canada). Cholestanol (Cat. no. D6128) (95%), α-tocopherol (Cat. no. 47783) (99.9%), γ-tocopherol (Cat. no. 47785) (96.8%), δ-tocopherol (Cat. no. 47784) (94%), chloroform (Cat. no. 366927), methanol (LCMS grade, Cat. no. 106035), acetonitrile (LCMS grade, Cat. no. 100029), hexane (270504) and formic acid (Cat. no. 33015) were purchased from Sigma Aldrich (Oakville, ON, Canada) while 3,4-dihydro-2-methyl-2-(4,8,12-trimethyltridecyl)-2H-1-benzopyran-6-ol (rac tocol), Cat. no. ab144067 (95%) was obtained from Abcam (Toronto, ON, Canada). Canola oil deodorizer distillate (CODD) was obtained from LDM Foods (Yorkton, SK, Canada).

### 3.2. Standard Solutions

Stock solution of reference standard phytosterols and tocopherols were prepared by dissolving each compound at 1 mg/mL in chloroform. A mixed stock solution containing all 7 standards (phytosterols and tocopherols) was prepared at 50 µg/mL by pipetting the required volume from the stock solution and diluting it with acetonitrile. Similarly, internal standards reference stock solution (cholestanol or d_6_-cholesterol for phytosterols and rac tocol for tocopherols) were prepared by dissolving each at 1 mg/mL in chloroform. Note that two different internal standards for phytosterols were evaluated during method development and d_6_-cholesterol was eventually chosen as it did not cause interferences (see results and discussion). A mixed internal standard working solution of rac tocol and cholestanol or d_6_-cholesterol was prepared by pipetting the required volume of each analyte and diluting it with acetonitrile for a final concentration of 100 µg/mL. Calibration standards in the concentration range 0.05–10 µg/mL were prepared in triplicates in acetonitrile. All internal standards were spiked into each calibrant at a final concentration of 2.5 µg/mL. Gamma and beta tocopherols are positional isomers and were not chromatographically resolved. As such, they are quantified collectively, as previously done using gamma tocopherol as reference standard [39,58,59,60].

### 3.3. Sample Preparation

#### Unsaponifiable Matter

For the preparation of the unsaponifiable matter that contains both phytosterols and tocopherols, previously developed extraction method was adapted [40]. Briefly, 5 g of CODD was saponified with 1 M KOH in 95% ethanol for 1 h at 65 °C. The mixture was cooled to room temperature and 50 mL of distilled water was added. Unsaponifiables were extracted three times with 50 mL hexane and the combined organic phase was washed with 10% EtOH to remove excess KOH until the washings were neutral to phenolphthalein. The organic phase was dried by passing it through anhydrous sodium sulfate followed by evaporation of hexane using a Buchi rotary evaporator R200 (Buchi corp., DE, USA). The residue was further dried under high vacuum using Trivac vacuum D4A (Leybold vacuum products Inc., Export, PA, USA) overnight.

### 3.4. HPLC and MS Parameters

Chromatographic separation of the analytes was performed on an Agilent Acquity UPLC (Agilent Technologies, Mississauga, ON, Canada) system with an Agilent Poroshell guard column (2.1 mm × 5 mm, 2.7 µm (Cat. no. 821725-911) and 2.1 mm × 5 mm, 1.9 µm (Cat. no. 821725-940). Flow rate was investigated in the range 150–800 µL/min and the run times ranged 1.3–2 min. The column temperature was also evaluated in the range 10–20 °C, with better separation achieved at 10 °C. An isocratic elution of acetonitrile: methanol (99:1 *v*/*v*) with 0.03% acetic acid was used at a flow rate of 200 µL/min and 600 µL/min for 2.7 µm and 1.9 µm guard column, respectively, and the injection volume was 1.0 µL.

Detection and quantification were performed using an AB Sciex 6500 QTRAP^®^ quadruple-linear ion trap (QqQ-LIT) mass spectrometer equipped with an atmospheric pressure chemical ionization (APCI) source (AB Sciex, Concord, ON, Canada). The analysis was performed by applying basic scheduled MRM algorithm, and two transitions (quantifier and qualifier) were monitored for each compound (Table 2). Tandem mass spectrometric analysis (MS/MS) using APCI was employed in positive ionization as recently optimized [39]. The following interface parameters were used: source temperature 380 °C, curtain gas 25 psi, nebulizer current 2.5 µA, and an ion source gas1 30 psi. Collision energy (CE) and declustering potential (DP) of each transition were optimized by direct infusion of individual reference standards.

Both instrument control and data acquisition were done using Analyst software 1.7 while data processing and quantification was done using MultiQuant 3.0.3 and applying SignalFinder integration algorithm. The algorithm SignalFinder is based on peak modelling that allows for better integration of poorly resolved peaks. This leads to consistent integration of peaks of interest across the concentration range. Due to the short analysis time, a scheduled MRM (sMRM) was adopted to promote reliability by ensuring enough data points were collected across the chromatographic peak. The target scan time (t_Target_) (i.e., cycle time) was optimized to ensure at least 10 data points across a chromatographic peak were acquired for accurate quantification. A t_Target_ values in the range 0.3–0.6 s were evaluated and the value with at least 10 data points and acceptable peak shape was chosen as optimal. To ensure complete monitoring of all transitions, a detection window of 35 s was chosen. The mass transitions, CE, DP, and retention times of all target compounds including the internal standards are shown in Table 2.

### 3.5. Method Validation

The International Council for Harmonization of Technical Requirements for Pharmaceuticals for Human Use (ICH) guidelines [55] were used to ensure the validity of the method, including linearity, sensitivity, accuracy, precision, repeatability, matrix effects, recovery, and stability. A key challenge, however, is the lack of an analyte free matrix. Both the calibration standards and quality control (QC) samples were prepared in a surrogate matrix that is a neat solvent, namely acetonitrile. A blank matrix containing very low level of endogenous analyte can be used to prepare QC samples during method validation for endogenous metabolites [56]. However, in this study, the concentration of the majority of the analytes were above the middle of the linear range; hence, the choice for a surrogate matrix approach. Exceptions were delta tocopherol (not detected in the matrix) and stigmasterol which was present at relatively low concentrations. Thus, in addition to validation using QCs prepared in neat solvent, both delta tocopherol and stigmasterol QC samples were prepared in the matrix and evaluated, as described in Section 3.5.2.

#### 3.5.1. Calibration Curve and Sensitivity

A standard curve for each analyte with at least eight data points in the range 0.05–10 µg/mL was constructed by plotting the peak area ratios (peak area of analyte to that of internal standard) versus the analyte concentration. A least-square regression model with a weighting factor of 1/x was applied. The performance of the standard curve was assessed using coefficient of determination (r^2^) and by evaluating the deviation of standards from nominal concentration. Calibration curve is only accepted when the data points in the curve lies within ±15% relative standard deviation of nominal value except for lower limit of quantitation (LLOQ) that can be ±20%.

Limit of detection (LOD) was determined based on a signal to noise (S/N) ratio ≥ 3 while LLOQ was set at the lowest concentration that showed accuracy and precision within ±20% CV of the nominal value.

#### 3.5.2. Intraday and Interday Accuracy and Precision

To establish intraday and interday accuracy and precision, quality control samples at four concentration levels ((LLOQ, low quality control (LQC), middle quality control (MQC), and high-quality control (HQC)) were analyzed. The LQC was within 3-fold of the LLOQ, the MQC was at the middle point of the standard curve range, and the HQC was within 80% of the upper limit of quantitation (ULOQ). In the case of stigmasterol, the endogenous amount was first determined and MQC and HQC prepared by spiking the matrix with appropriate amount of the standard. The same approach was applied in the case of delta tocopherol; however, it was not detected in the matrix and all QC samples were prepared in the matrix. Accuracy and precision for QC samples should lie within ± 15% CV of nominal value except for LLOQ, which can be within ±20% CV. Intraday accuracy and precision were calculated using QC replicates (*n* = 4) in a single analytical run whereas interday accuracy and precision measurements were conducted using QC replicates (*n* = 4) prepared on three consecutive days.

#### 3.5.3. Recovery and Matrix Effect

For analyte recovery, determination was assessed in the unsaponifiable matter. Quality controls (LQC, MQC, and HQC) were spiked into the unsaponifiable matter and the analyte relative recovery was calculated as shown in Equation (1). Since there are no clear guidelines for recovery experiments for endogenous metabolites where analyte free matrix is not available, the described approach for the determination of recovery was adapted. The endogenous concentration in the matrix was first measured and then QC standards were spiked.

(1) $$\mathrm{Recovery}\;\left(\%\right)\;=\;\frac{\mathrm{Csample}\;\mathrm{spiked-Csample}}{\mathrm{Cspike}\;\mathrm{level}}\;\times \;100$$

where C_sample spiked_ is the measured concentration in spiked sample after extraction, C_sample_ is the endogenous concentration in un-spiked sample and C_spike level_ is the spiked concentration.

For matrix effect, Equation (2) was applied and evaluation was conducted by spiking the unsaponifiable matter at two concentration levels, LQC and HQC. The spiked amount was equivalent to that amount that would bring the QC to the required concentration with the endogenous amount in consideration.

(2) $$\mathrm{Matrix}\;\mathrm{effect}\;\left(\%\right)\;=\;\frac{\mathrm{Asample}\;\mathrm{spike}}{\mathrm{Aneat}}\;\times \;100$$

where A_sample spike_ is peak area of spiked sample, and A_neat_ is peak area of the same amount in neat solvent.

#### 3.5.4. Stability

Benchtop and autosampler stability were evaluated using QC samples prepared in the matrix. δ-tocopherol at LLOQ, LQC, MQC, and HQC and stigmasterol at MQC, and HQC were employed in this study. Benchtop QCs were left at room temperature for 12 h while autosampler were kept in the autosampler at 10 °C for 12 h before analysis.

## 4. Application of the Method for the Analysis of Phytosterols and Tocopherols

The validated method was applied for the determination of phytosterols and tocopherols present in canola oil deodorizer distillate (CODD). CODD is a waste stream from canola oil refining process and is rich in phytosterols and tocopherols. Both phytosterols and tocopherols are present in the unsaponifiable fraction. The unsaponifiable matter of the CODD have been used for the extraction of bioactive metabolites [61] and it is important to measure the concentration of target analytes prior to extraction. For LC-MS/MS analysis, 1 mg/mL of sample was prepared in chloroform and 50 µL was pipetted into an amber HPLC vial, internal standards added, and the contents were diluted to 1 mL with acetonitrile.

## 5. Conclusions

A fast chromatography-tandem mass spectrometric method (FC-MS/MS) was developed for the quantitative analysis of phytosterols and tocopherols in vegetable oil samples. The reported approach addressed the shortcomings of FIA-MS, where analyte-to-analyte interferences is a major concern. We have shown that application of a guard column with smaller particle size is ideal and substantially shortens the run time. The method has a total run time of 1.3 min. Good separation of all the analytes including the ability to differentiate the interfering peaks were achieved. The method was successfully validated and applied in the quantitative determination of phytosterols and tocopherols in CODD. In addition, this method can be used as starting point for the development of qualitative and quantitative analysis of the purified phytosterols and tocopherols, after extracting them from the unsaponifiable matter. This analytical method can also be applied for the quantification of phytosterols and tocopherols in other vegetable oil sources, such as corn, sunflower, soybean, and olive. It can also be the basis for the development of quantitative methods in pharmaceutical formulations, supplements, and beverages. However, appropriate sample preparation should be adopted, and the method will require optimization for the intended purpose.

## Figures and Tables

**Table 1:** Observed analyte-to-analyte interferences amongst phytosterols due to the presence of other minor ions.

Compound	Molecular Weight	^a^ [M + H − H_2_O]^+^ *m*/*z*	[M + H − 4H]^+^ *m*/*z*	[M + H − 2H]^+^ *m*/*z*
Stigmasterol	412.7	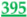	409	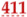
Brassicasterol	398.7	381	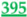	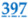
β-sitosterol	414.7	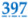	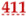	413
Campesterol	400.7	383	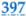	399
Cholestanol (IS)	388.7	371	385	387
d_6_-cholesterol (IS)	392.7	375	389	391

^a^ [M + H − H_2_O]^+^ is the ion used for quantification.

**Table 2:** The mass transitions, collision energy (CE), and retention times of all target compounds including the internal standards. Declustering potential (DP) of 40 V was applied to all analytes.

Compound		Retention Time (min)	Precursor Ion (*m/z*)	MRM Transitions (*m/z*)	CE (V)
Phytosterols	Brassicasterol	0.50	381.4	297.4	22 30
				147.1	
	Campesterol	0.61	383.4	161.1	30 30
				147.1	
	Stigmasterol	0.63	395.4	297.4	23 26
				83.2	
	β-Sitosterol	0.73	397.4	161.2	27 28
				134.9	
Tocopherols	Delta Tocopherol (δ)	0.32	402.4	177	32 38
				137	
	Gamma Tocopherol (γ)	0.38	416	151.1	37 34
				191.2	
	Alpha Tocopherol (α)	0.44	430	165.3	38 36
				205.2	
Internal Standards	D_6_-Cholesterol	0.51	375.4	152	30 28
				167	
	Cholestanol	0.95	371.4	95	32 30
				109	
	Rac Tocol	0.26	388.4	163.2	30
				122	40

**Table 3:** Inter-day and intra-day accuracy and precision of phytosterols and tocopherols prepared in surrogate matrix ^a^ and sample matrix ^b^ (*n* = 4).

Compound *^a^*		Concentration (µg/mL)	Intra-day		Inter-day 1		Inter-day 2		Inter-day 3	
			Accuracy (%)	Precision (%RSD)	Accuracy (%)	Precision (%RSD)	Accuracy (%)	Precision (%RSD)	Accuracy (%)	Precision (%RSD)
δ-Tocopherol	LLOQC	0.25	97.83	6.06	92.86	3.96	95.05	5.42	96.85	3.45
	LQC	0.75	94.19	4.73	90.96	4.59	96.08	0.85	102.59	5.90
	MQC	5.5	101.08	1.82	94.46	3.86	96.59	2.51	95.70	1.13
	HQC	8	98.16	5.37	96.08	3.14	99.42	2.63	99.23	3.64
β/γ-Tocopherol	LLOQC	0.25	107.29	2.93	88.74	0.72	94.80	3.68	100.43	3.22
	LQC	0.75	96.04	2.62	90.64	4.39	94.34	1.70	94.20	3.50
	MQC	5.5	100.00	4.97	90.30	4.32	96.68	3.86	95.67	2.35
	HQC	8	98.01	2.57	90.49	3.62	99.49	1.46	100.35	2.77
α-Tocopherol	LLOQC	0.25	118.38	0.49	109.49	2.32	118.32	2.29	115.59	1.70
	LQC	0.75	94.22	3.34	89.46	3.51	91.76	1.65	94.58	4.65
	MQC	5.5	104.32	2.78	92.00	2.82	95.14	2.86	99.50	1.92
	HQC	8	102.69	4.83	96.42	2.02	102.74	3.43	103.81	2.86
Brassicasterol	LLOQC	0.05	93.91	9.66	99.01	9.66	109.43	6.81	117.57	3.43
	LQC	0.15	97.14	7.50	98.98	7.50	102.26	1.07	96.47	2.70
	MQC	5.5	96.75	1.80	96.65	1.80	97.06	3.97	95.72	0.72
	HQC	8	95.91	3.00	101.17	3.00	99.55	1.83	101.44	3.73
Campesterol	LLOQC	0.05	99.39	0.67	114.21	5.57	104.15	2.88	108.77	1.37
	LQC	0.15	101.60	3.01	106.84	3.52	10.3.9	0.77	102.95	2.93
	MQC	5.5	101.77	1.68	102.33	1.37	103.91	2.95	97.43	4.10
	HQC	8	97.57	1.24	102.37	5.16	101.16	3.35	98.53	2.80
Stigmasterol	LLOQC	0.05	94.56	0.96	113.98	8.81	116.74	3.80	118.44	4.26
	LQC	0.15	94.00	3.38	103.28	6.18	103.93	1.25	102.48	2.52
	MQC	5.5	96.72	2.47	95.68	2.42	98.97	1.82	95.05	4.09
	HQC	8	94.75	2.83	97.21	6.27	98.19	4.36	95.32	3.82
β-Sitosterol	LLOQC	0.05	99.28	2.86	108.12	3.23	103.45	5.96	110.09	3.55
	LQC	0.15	100.18	2.65	108.53	4.56	107.89	2.88	102.19	2.36
	MQC	5.5	102.31	0.87	99.72	1.73	103.56	1.66	97.56	3.81
	HQC	8	98.92	1.82	100.75	4.24	103.31	2.94	99.01	2.95
**Compound** *^**b**^*		**Concentration (µg/mL)**	**Intra-Day**		**Inter-Day 1**		**Inter-Day 2**		**Inter-Day 3**	
			**Accuracy (%)**	**Precision (%RSD)**	**Accuracy (%)**	**Precision (%RSD)**	**Accuracy (%)**	**Precision (%RSD)**	**Accuracy (%)**	**Precision (%RSD)**
δ-Tocopherol	LLOQC	0.25	87.4	1.08	113.57	3.84	106.18	5.49	117.17	2.23
	LQC	0.75	96.4	1.62	103.04	3.82	96.81	0.63	108.50	4.29
	MQC	5.5	94.1	1.97	97.06	1.70	106.0	1.33	97.76	5.06
	HQC	8	99.9	3.25	96.59	3.91	91.20	1.40	91.99	2.67
Stigmasterol	MQC	5.5	93.3	3.63	95.95	0.05	98.38	4.59	99.32	7.63
	HQC	8	96.5	2.42	97.11	1.94	103.02	3.91	103.37	0.87

^a^ Surrogate matrix used was neat solvent (acetonitrile); ^b^ Sample matrix is CODD extract.

**Table 4:** Matrix effect and recovery studies for delta tocopherol and stigmasterol.

Concentration (µg/mL)	Delta Tocopherol		Stigmasterol	
	Matrix Effect	%Recovery	Matrix Effect	%Recovery
0.75	108.22 ± 0.96	104.60 ± 4.05	-	-
5.5	-	99.94 ± 1.68	-	95.49 ± 0.06
8	93.96 ± 2.1	98.76 ± 3.72	106.74 ± 5.8	96.89 ± 2.03

**Table 5:** Phytosterols and tocopherols content (%) in CODD.

	Hot Pressed CODD	Hot Pressed CODD	Cold Pressed CODD
	Batch 1	Batch 2	
Gamma tocopherol	2.20 ± 0.02	1.26 ± 0.14	2.47 ± 0.17
Alpha tocopherol	0.90 ± 0.01	0.63 ± 0.01	1.43 ± 0.01
Delta tocopherol	nd	nd	nd
Brassicasterol	7.59 ± 0.13	7.10 ± 0.14	6.57 ± 0.21
Campesterol	11.24 ± 0.51	9.75 ± 0.46	11.93 ± 0.27
Stigmasterol	1.08 ± 0.10	1.00 ± 0.03	1.13 ± 0.18
β-Sitosterol	14.73 ± 0.31	12.03 ± 0.46	15.68 ± 0.59

nd—not detected.

## Data Availability

The data presented in this study are available in the above manuscript and its supporting information.

## Supplementary Materials

The following are available online. Table S1: Summary for some reported studies outlining analyte-to-analyte interferences for the analysis of plant sterols, Table S2: Benchtop and autosampler stability in matrix spiked QCs shown as mean ± SD, Figure S1: Cholestanol as an internal standard showed analyte interferences, where insert chromatograms shown by → is extracted ion chromatogram for brassicasterol and → for campesterol, Figure S2: Cholestanol interfering ion at *m*/*z* 383 shows a similar MS/MS spectrum as that of campesterol monitored ion [M + H − H_2_O]^+^ at *m*/*z* 383, Figure S3: Brassicasterol MS/MS of interfering ion [M + H − 4H]^+^ at *m*/*z* 395 showing similar MS/MS spectrum as that of Stigmasterol monitored ion [M + H − H_2_O]^+^ at *m*/*z* 395, Figure S4: Campesterol MS/MS of interfering ion [M + H − 4H]^+^ at *m*/*z* 397 showing similar MS/MS spectrum as that of β-sitosterol monitored ion [M + H − H_2_O]^+^ at *m*/*z* 397, Figure S5: Merging of the interfering peak from campesterol *m*/*z* 397→161/135 with β-sitosterol peak at high concentration (using 2.7 µm guard column), Figure S6: Campesterol (*m*/*z* 383→161/147) interference peak (insert) from d_6_-cholesterol, Figure S7: Interfering peaks are distinguishable at both low (A1, A2) and high (B1, B2) concentration when 1.9 µm guard column was employed, Figure S8: Calibration curves for tocopherols (A) and phytosterols (B).

## Sample Availability

Samples of the compounds are available from the authors.
