# Supplementary material for: Novel Fast Chromatography-Tandem Mass Spectrometric Quantitative Approach for the Determination of Plant-Extracted Phytosterols and Tocopherols
Source: Molecules. 2021 Mar 5;26(5):1402. doi: 10.3390/molecules26051402 (PMC7961602; doi:10.3390/molecules26051402)
Supplement: Supplementary file 1 [file molecules-26-01402-s001.pdf]

Novel Fast Chromatography-Tandem Mass Spectrometry Quantitative Approach for the Determination of Plant-Extracted Phytosterols and Tocopherols.

George Gachumi[1], Alice Demelenne[2], Asmita Poudel[1], Zafer Bashi[3], and Anas El-Aneed[1*].

[1]Drug Design and Discovery Group, College of Pharmacy and Nutrition, University of Saskatchewan, Saskatoon, SK, S7N 5E5, Canada

[2]Laboratory for the Analysis of Medicines, Department of Pharmacy, CIRM, University of Liège, Belgium

Supplementary materials

Table S1: Summary for some reported studies outlining analyte-to-analyte interferences for the analysis of plant sterols.

Analyte	$[M + H – H_2O]^+$ *m/z*	MS Condition	*m/z* value of corresponding interference ion	Reference
campesterol	383	APCI (+)-MS/MS	395 matching $[M + H – H_2O]^+$ of stigmasterol and,<br>397 matching $[M + H – H_2O]^+$ of sitosterol	[1]<br>[1,2]
sitosterol	397	APCI (+)-MS/MS	409 matching $[M + H – H_2O]^+$ of cycloartenol	[1]
stigmasterol	395	APCI (+)-MS<br>APCI (+)-MS/MS	383 matching $[M + H – H_2O]^+$ of campesterol and,<br>409 matching $[M + H – H_2O]^+$ of cycloartenol	[3]<br>[2]

Table S2. Benchtop and autosampler stability in matrix spiked QCs shown as mean ± SD.

Concentration (μg/mL)	Delta tocopherol		Stigmasterol	
	Benchtop	Autosampler	Benchtop	Autosampler
0.25	102.70 ± 2.72	110.75 ± 1.06	-	-
0.75	106.91 ± 1.29	102.68 ± 5.85	-	-
5.5	92.81 ± 3.10	101.25 ± 2.62	91.50 ± 2.12	97.23 ± 1.73
8	101.25 ± 2.62	100.23 ± 5.01	104.50 ± 0.71	98.50 ± 2.12

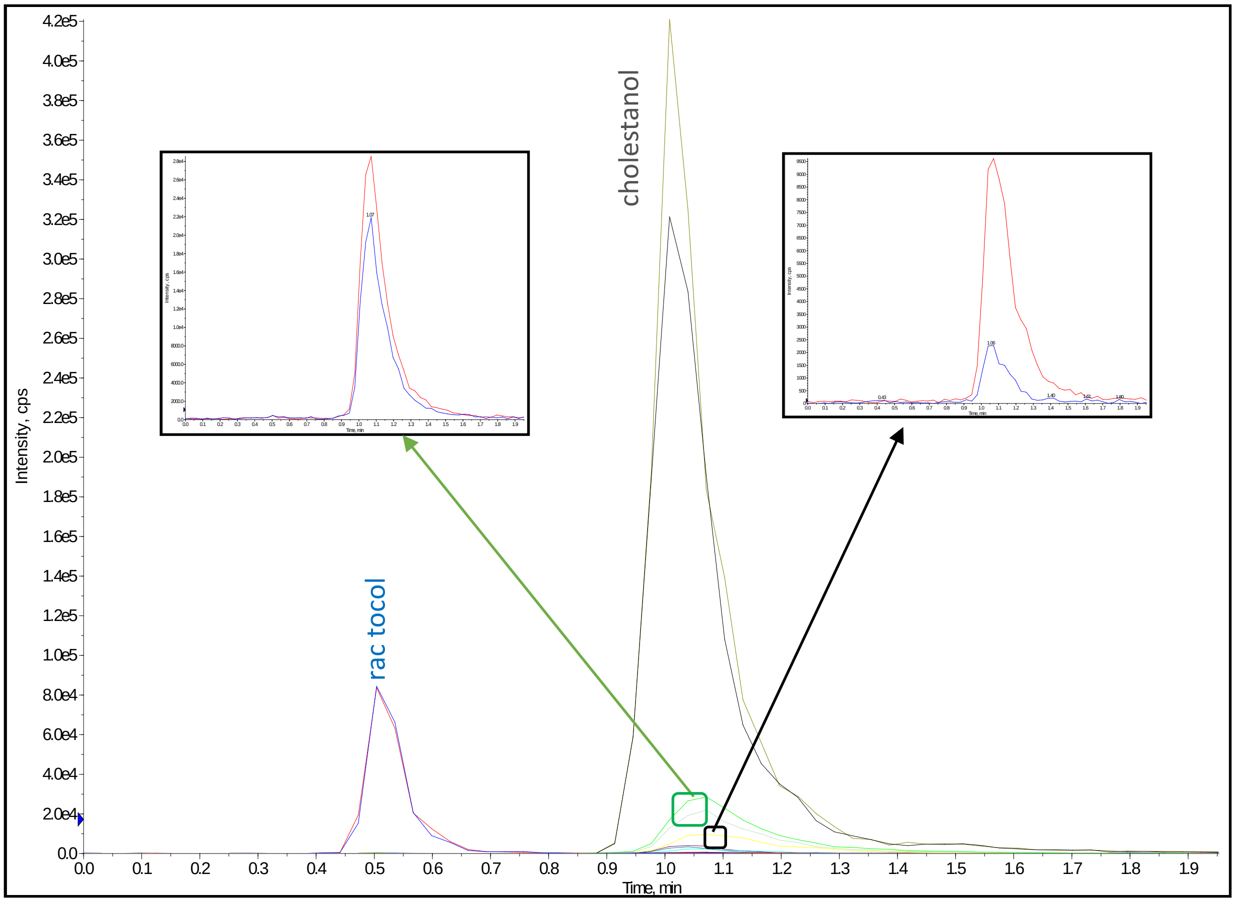

Figure S1. Cholestanol as an internal standard showed analyte interferences, where insert chromatograms shown by → is extracted ion chromatogram for brassicasterol and → for campesterol.

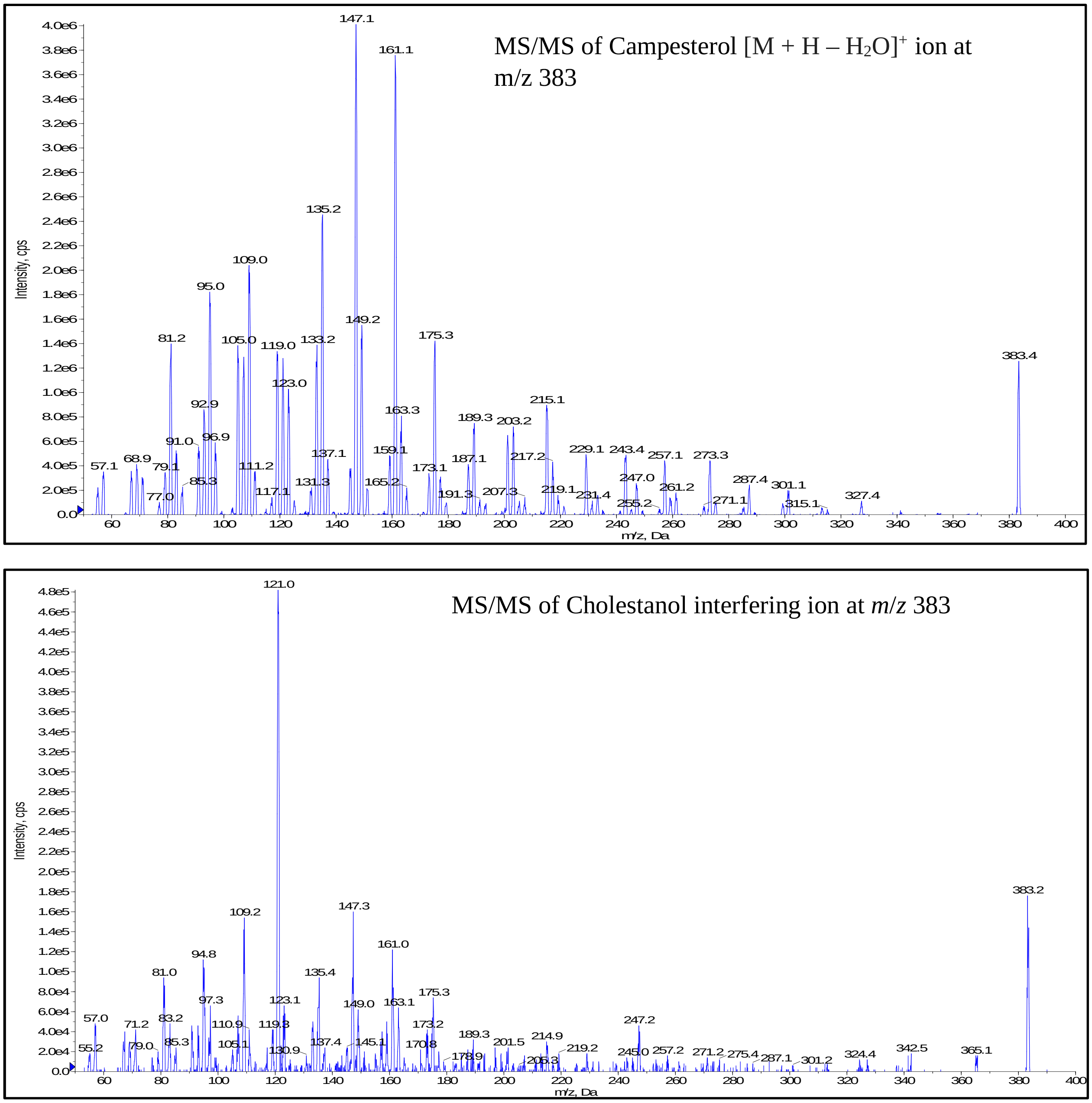

Figure S2. Cholestanol interfering ion at *m*/*z* 383 shows a similar MS/MS spectrum as that of campesterol monitored ion $[M + H - H_2O]^+$ at *m*/*z* 383

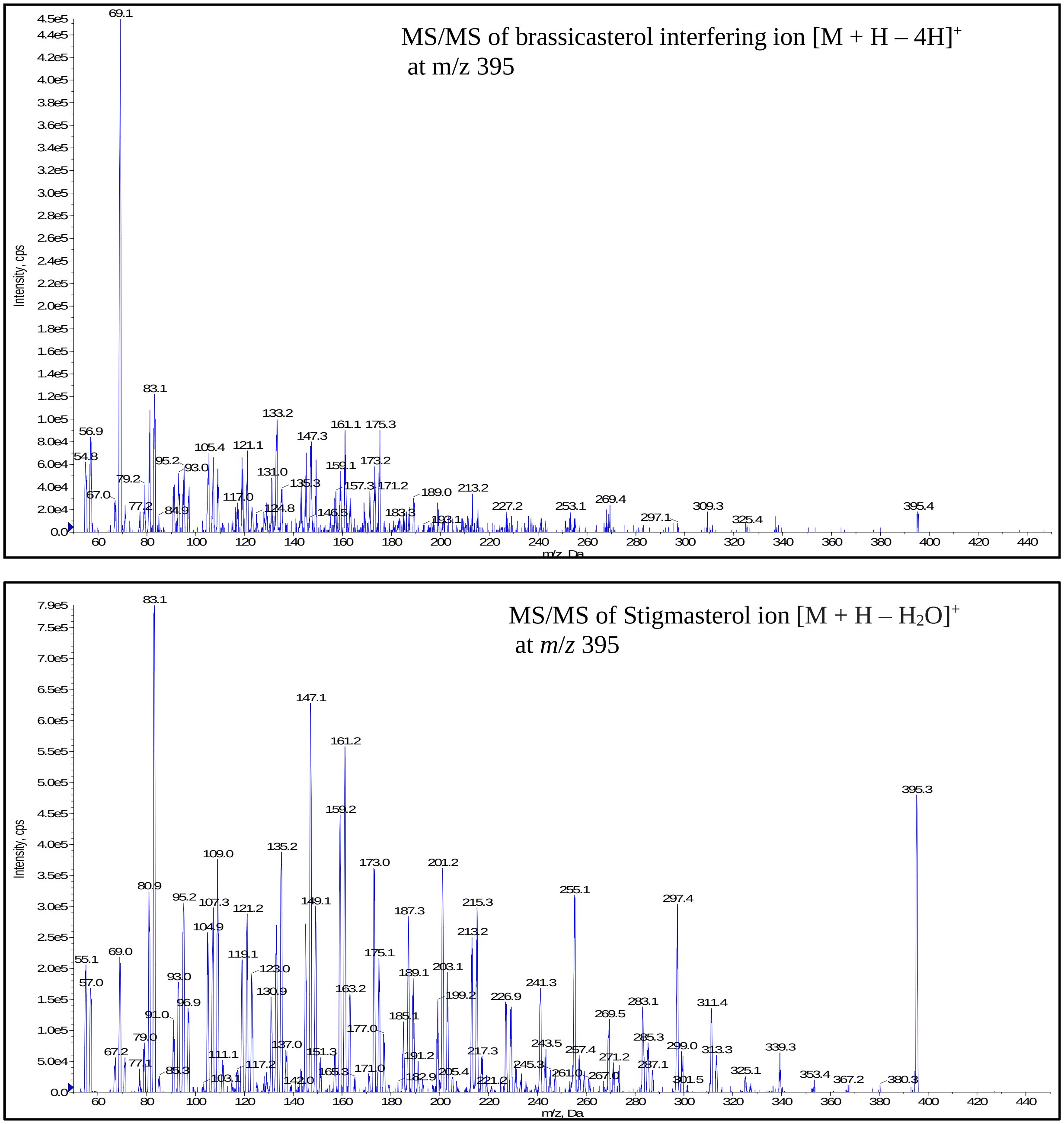

Figure S3. Brassicasterol MS/MS of interfering ion $[M + H – 4H]^+$ at *m*/*z* 395 showing similar MS/MS spectrum as that of Stigmasterol monitored ion $[M + H – H_2O]^+$ at *m*/*z* 395.

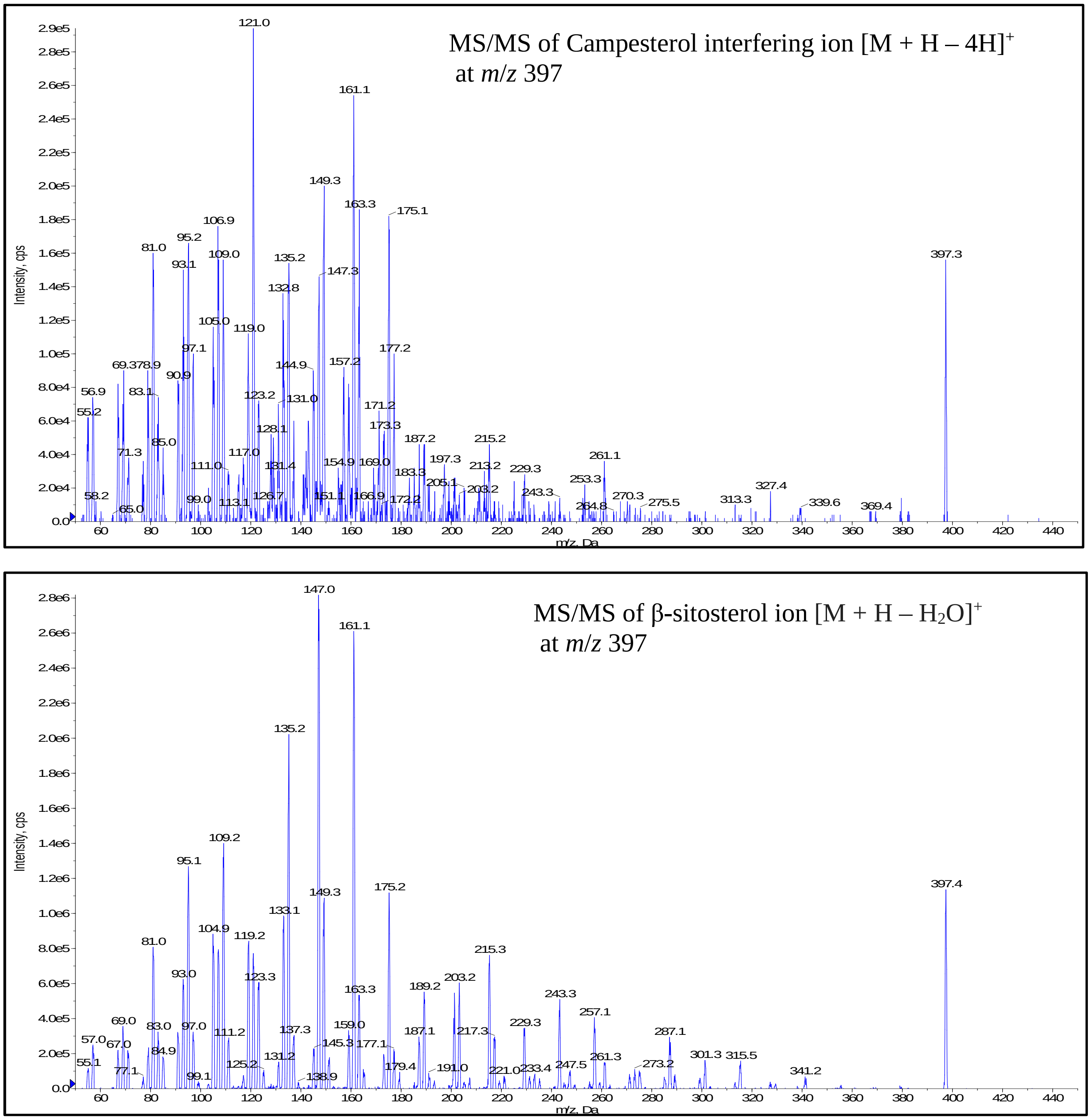

Figure S4. Campesterol MS/MS of interfering ion [M + H – 4H]$^+$ at m/z 397 showing similar MS/MS spectrum as that of β-sitosterol monitored ion [M + H – H_2O]$^+$ at m/z 397.

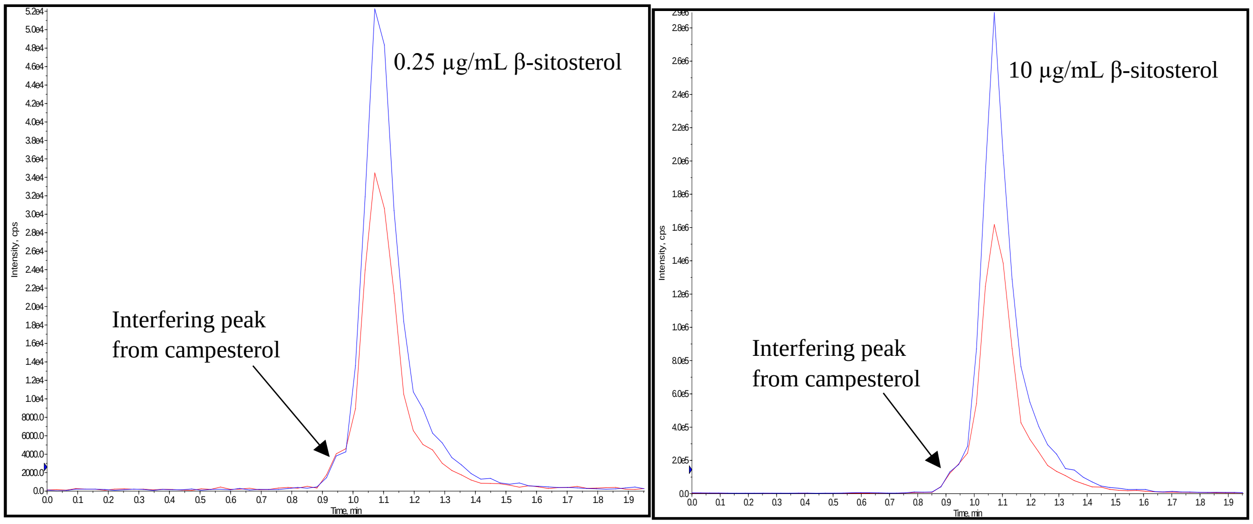

Figure S5. Merging of the interfering peak from campesterol *m/z* 397→161/135 with β-sitosterol peak at high concentration (using 2.7 µm guard column).

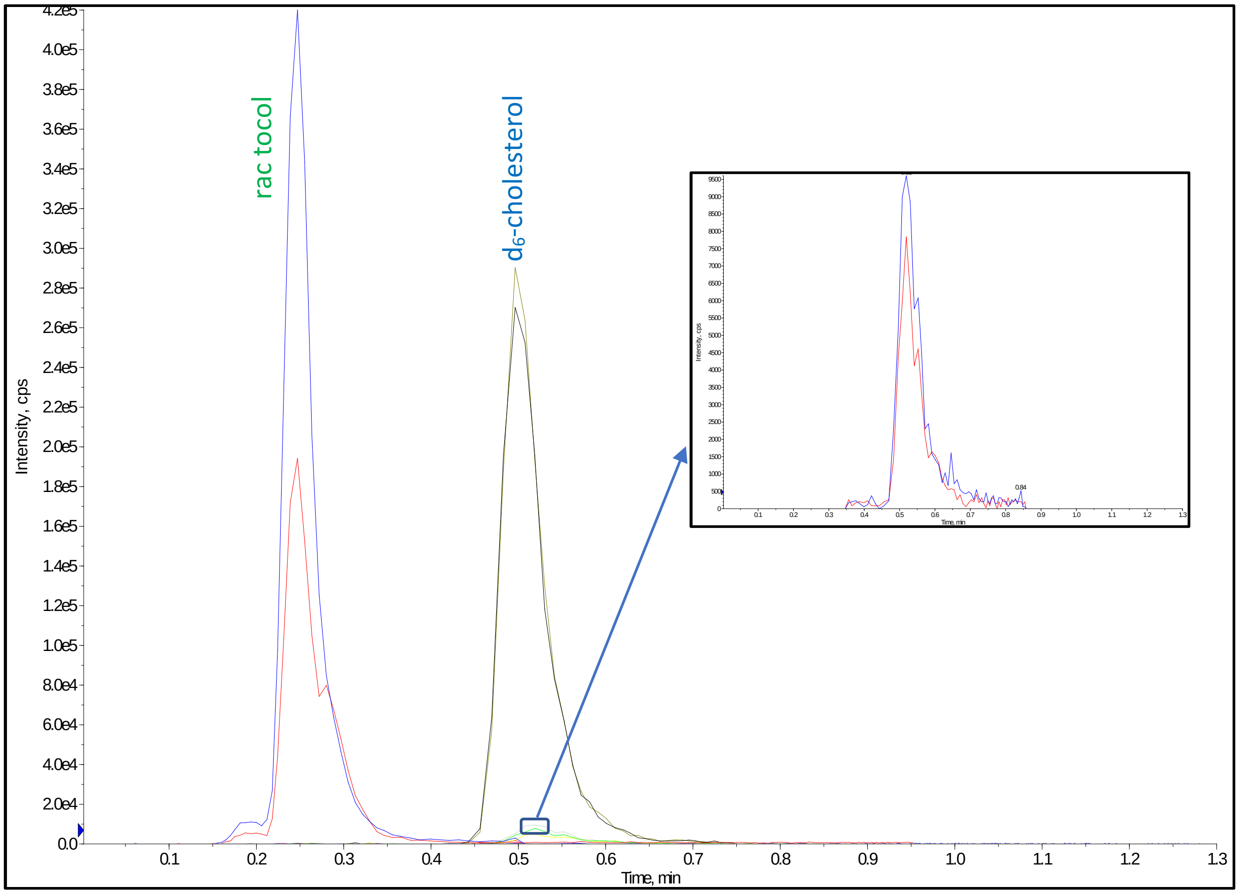

Figure S6. Campesterol (*m*/*z* 383→161/147) interference peak (insert) from d_6-cholesterol.

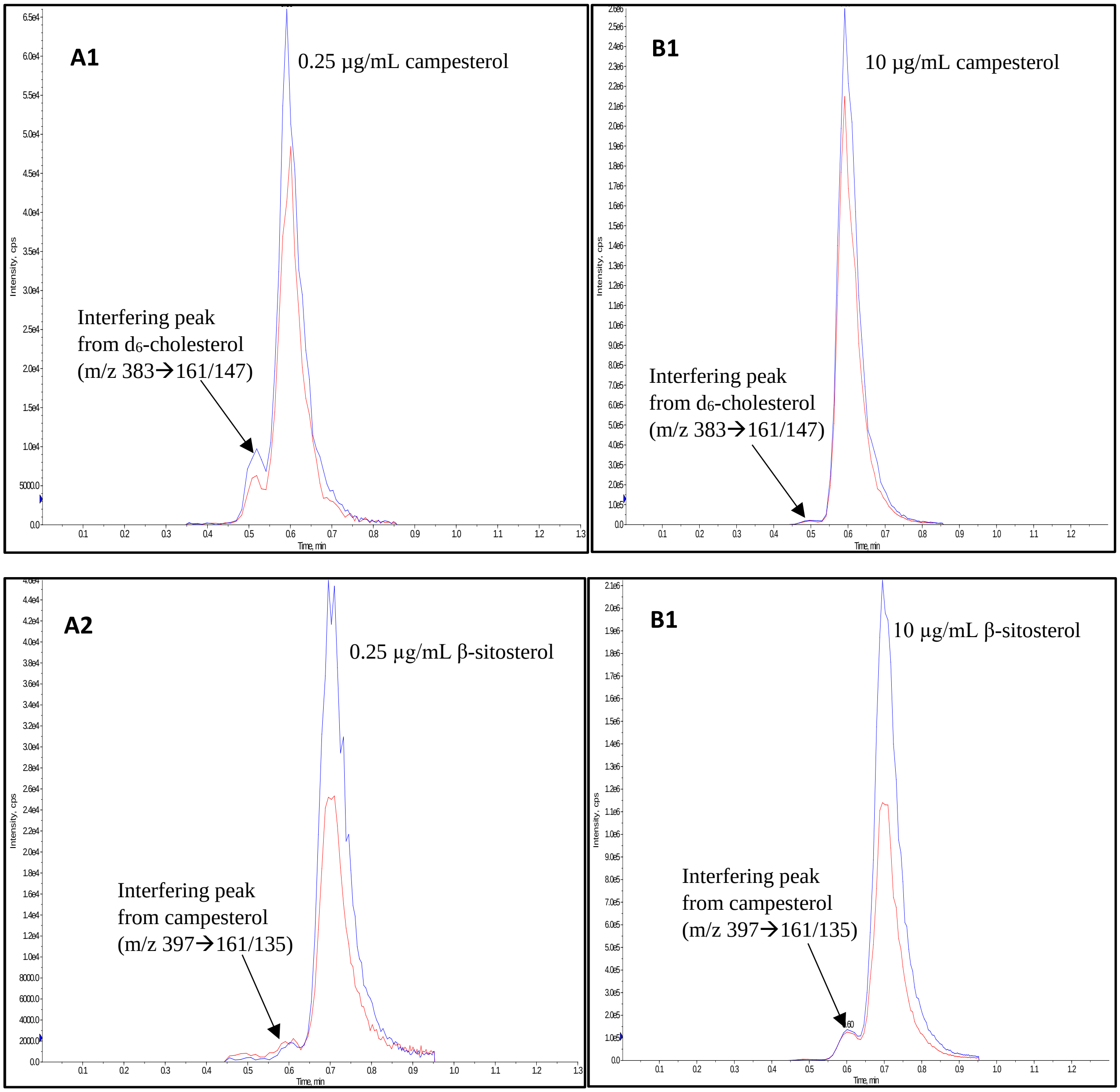

Figure S7. Interfering peaks are distinguishable at both low (A1, A2) and high (B1, B2) concentration when 1.9 µm guard column was employed.

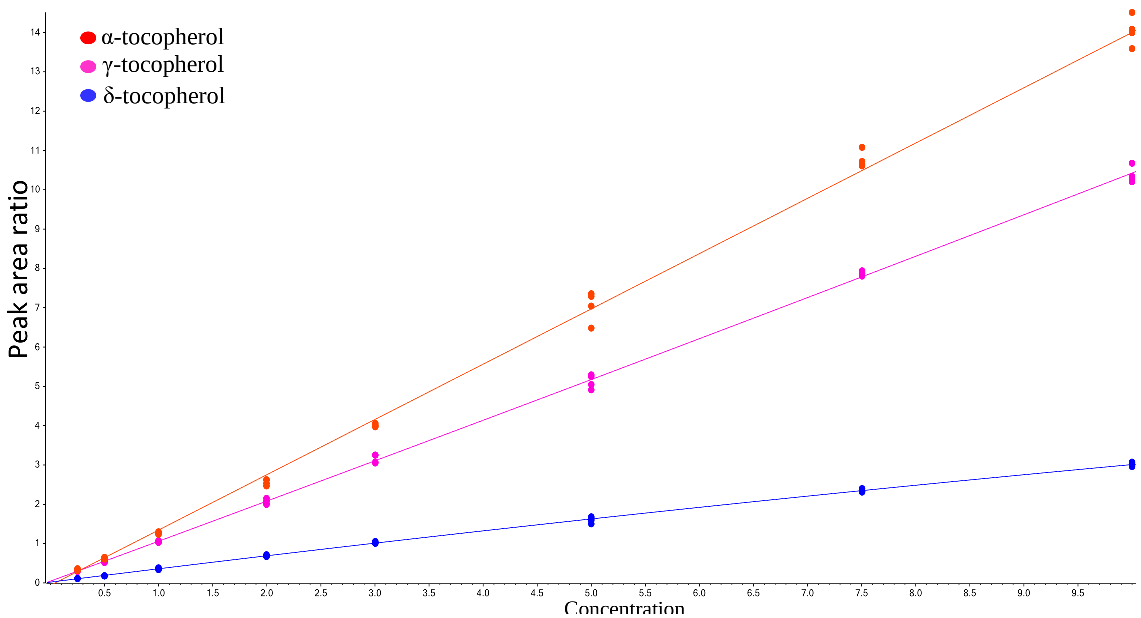

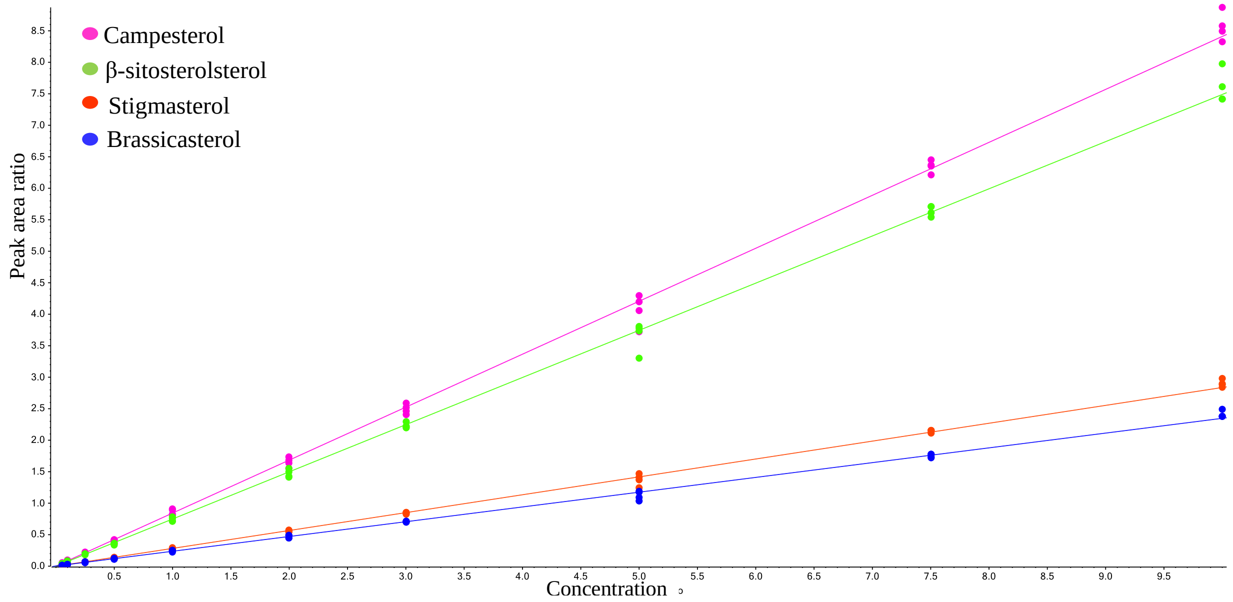

Figure S8. Calibration curves for tocopherols (A) and phytosterols (B).

References.

1. Bedner, M.; Schantz, M.M.; Sander, L.C.; Sharpless, K.E. Development of liquid chromatographic methods for the determination of phytosterols in standard reference materials containing saw palmetto. *J. Chromatogr. A* **2008**, *1192*, 74-80.
2. Rozenberg, R.; Ruibal-Mendieta, N.L.; Petitjean, G.; Cani, P.; Delacroix, D.L.; Delzenne, N.M.; Meurens, M.; Quetin-Leclercq, J.; Habib-Jiwan, J.-L. Phytosterol analysis and characterization in spelt (triticum aestivum ssp. Spelta l.) and wheat (t. Aestivum l.) lipids by lc/apci-ms. *Journal of cereal science* **2003**, *38*, 189-197.
3. Ishida, N. A method for simultaneous analysis of phytosterols and phytosterol esters in tobacco leaves using non aqueous reversed phase chromatography and atmospheric pressure chemical ionization mass spectrometry detector. *J. Chromatogr. A* **2014**, *1340*, 99-108.
